# It‘s Complicated: Why Are There So Few Commercially Successful Crop Varieties Engineered for Disease Resistance?

**DOI:** 10.1111/mpp.70077

**Published:** 2025-03-19

**Authors:** Peter Balint‐Kurti, Qingli Liu

**Affiliations:** ^1^ Plant Science Research Unit USDA‐ARS Raleigh NC USA; ^2^ Department of Entomology and Plant Pathology North Carolina State University Raleigh NC USA; ^3^ Seeds Research, Syngenta Crop Protection LLC Research Triangle Park Durnham NC USA

**Keywords:** disease resistance, genetic engineering, GMO

## Abstract

It is more than 40 years since the era of transgenic plants began and more than 30 years after the cloning of the first plant disease resistance genes. Despite extensive progress in our mechanistic understanding and despite considerable sustained efforts in the commercial, nonprofit, academic and governmental sectors, the prospect of commercially viable plant varieties carrying disease resistance traits endowed by biotechnological approaches remains elusive. The cost of complying with the regulations governing the release of transgenic plants is often cited as the main reason for this lack of success. While this is undeniably a substantial hurdle, other transgenic traits *have* been successfully commercialised. We argue that a significant portion of the challenges of producing crop varieties engineered for disease resistance is intrinsic to the trait itself. In this review, we briefly discuss the main approaches used to engineer plant disease resistance. We further discuss possible reasons why they have not been successful in a commercial context and, finally, we try to derive some lessons to apply to future efforts.

## 1 Introduction

Harold Flor pioneered the investigation of plant–pathogen genetics in the 1940s with his formulation of the gene‐for‐gene concept (Flor 1942), in which he demonstrated that specific dominant *Resistance* (or *R‐*) genes in the host corresponded to specific *Avirulence* (*Avr*) genes in the pathogen. If both corresponding genes were present in an interaction, the result was pathogen recognition and the triggering of a defence response that resulted in resistance. If either gene was absent, there was no recognition and the result was that the plant was susceptible, and disease progressed. This concept proved to be widely applicable and has since served as the main organising principle behind much of the subsequent work in the genetics of molecular plant–microbe interactions field (Gassmann and Bhattacharjee 2012; Dodds 2023).

The era of transgenic plants, that is the era in which it was possible to express foreign genes in functional whole plants, could be considered to have begun around 1984 when *Nicotiana* plants expressing bacterial resistance genes were regenerated and shown to be able to transmit transgenically derived traits to their offspring in a Mendelian manner (De Block et al. 1984; Herrera‐Estrella et al. 2004). The field of molecular plant pathology also saw an important milestone in 1984 with the first molecular identification of an *Avr* gene, *AvrA* from *Pseudomonas syringae* (Staskawicz et al. 1984; Staskawicz 2009). Another milestone, the identification of the first major plant *R*‐gene, *Hm1*, occurred in 1992 (Johal and Briggs 1992). *Hm1* did not act as a typical ‘gene‐for‐gene’ *R*‐gene because it had a detoxification rather than a recognition function. However, many ‘canonical’ plant recognition‐type *R*‐genes were identified soon afterwards (Martin et al. 1993; Bent et al. 1994; Jones et al. 1994; Mindrinos et al. 1994; Whitham et al. 1994; Song et al. 1995). Current sequencing and bioinformatic techniques now make *R*‐gene identification a relatively straightforward process (Zhang et al. 2020a).

Numerous other advances in our knowledge of the molecular basis of plant–pathogen interactions have occurred in the intervening years. For example, it has been established that microbial pathogens introduce a suite of molecules (usually proteins), termed ‘effectors’, into the cell to facilitate the pathogenesis process and that, in most cases, Avr proteins are simply the class of these effectors that are recognised by R‐proteins. Consequently, resistance mediated by gene‐for‐gene mechanisms was termed ‘effector‐triggered immunity’ or ETI. Microbe‐derived molecules can also be recognised outside the cell. Usually, these molecules are not specifically associated with pathogenesis and are termed ‘pathogen‐associated molecular patterns’ or PAMPs. Membrane‐bound receptors, known as pattern recognition receptors (PRRs), recognise PAMPs in the apoplast and trigger a defence response known as pattern‐triggered immunity or PTI (Bent and Mackey 2007). In the last few years, it has emerged that ETI appears to function largely through amplifying and extending the PTI response (Rhodes et al. 2022). Many mechanisms that confer lower levels of resistance (or quantitative resistance) have also been defined. Some quantitative resistance mechanisms appear to function in similar ways to ETI and PTI, but many other mechanisms have been defined (Gou et al. 2023). It has become clear that plant disease resistance is controlled by a system of preformed and induced defences that interact in a complex and intimate way with other plant processes. For a summary of recent progress in our understanding of the functioning of plant defence systems, the reader is directed to several excellent reviews (e.g., Bent and Mackey 2007; Jones et al. 2016; Ngou et al. 2022; Gou et al. 2023; Dodds et al. 2024; Jones et al. 2024).

It is perhaps surprising that, despite this remarkable progress in our knowledge, there exist almost no commercially available plant varieties carrying biotech‐derived disease resistance traits. In fact, the most often‐cited success story in this area, that of the SunUp and UH Rainbow papaya varieties engineered to be resistant to papaya ringspot virus (Gonsalves 1998; Ferreira et al. 2002) was in a specialty crop and occurred more than 25 years ago. Other commercial releases of biotech disease resistance traits have been very scarce. In fact, the only examples we are aware of are several virus‐resistant squash varieties, also released in the 1990s, that use similar RNA interference (RNAi) technology (see below) to the aforementioned papaya varieties (Fuchs and Gonsalves 2007).

The various approaches used to engineer disease resistance in plants and their general lack of commercial success were recently the subject of a thorough review (Collinge and Sarrocco 2022; see table 1 in that paper). Here, we want to discuss why this profound progress in our understanding over the last 40 years, coupled with sustained, high‐quality and well‐funded efforts in the commercial, public and nonprofit sectors to apply this knowledge (Salmeron and Vernooij 1998; Dangl et al. 2013; Horvath 2018) has not yet led to more tangibly useful outcomes. We ask whether we can learn lessons from our sustained lack of success and whether we can apply them in our future efforts in this area.

We should also note that efforts to commercialise varieties engineered to improve many other traits, for example, nutritional enhancement, ripening control, processing, yield and abiotic stress resistance, have also not met with conspicuous success (Ricroch et al. 2022). This review specifically concerns efforts to engineer for disease resistance, so we will not consider these other traits further. While some of the issues discussed below are also pertinent for these other traits, others are specific to biotic stress.

## 2 A Brief History of Efforts to Engineer Plant Disease Resistance

Hundreds of papers have appeared in the academic literature that describe genes that have been introduced or manipulated by transgenic techniques and, latterly, by using gene editing, to make their plant hosts more resistant to one or multiple diseases. In the ‘early days’ four basic approaches to engineering resistance dominated:

- Resistance genes were transferred between species (e.g., Whitham et al. 1996; Tai et al. 1999; Narusaka et al. 2013).
- Overexpression of antimicrobial proteins or enzymes in defence pathways that resulted in the overproduction of antimicrobial metabolites (Brogue et al. 1991; Alexander et al. 1993; Carmona et al. 1993; Düring et al. 1993; Hain et al. 1993; Delaunois et al. 2009).
- Altered (usually increased) expression of endogenous defence response regulators (Cao et al. 1998) or of disease resistance genes (Laugé and De Wit 1998). While these approaches have been effective in conferring disease resistance, constitutive over‐induction of the defence response was often associated with reduced growth and yield (Heidel et al. 2004).
- Using RNAi mediated by small RNA (siRNA) molecules to suppress the expression of vital pathogen genes has been the only technique that yielded commercial varieties with effective resistance, though the mechanism by which resistance was achieved was not initially appreciated (Fuchs and Gonsalves 2007; Lindbo and Falk 2017).

In more recent years, various, more sophisticated, approaches, based on our increasingly nuanced understanding of the plant defence response system, have been proposed and demonstrated experimentally. Examples include

- As the mechanisms associated with RNAi have become better appreciated, siRNAs have been designed to suppress the expression of vital pathogen genes, thus reducing their pathogenesis. Various methods have been used to deliver the siRNAs to the pathogen. Host‐induced gene silencing (HIGS) uses transgenic expression of progenitor double‐stranded RNA molecules in the host to achieve this, while spray‐induced gene silencing (SIGS) uses direct application of siRNAs onto infected plants. These and other methods have been recently reviewed (Bilir et al. 2022; Beernink et al. 2024). Both HIGS (Urquhart et al. 2015) and SIGS (Stokstad 2024) techniques have been used to develop commercial varieties or treatments against insects, but not, so far, for microbial pathogens.
- Approaches that use transgenically introduced enzymes or transporters to digest or export pathogen toxins have shown promise in several systems (Zhang et al. 1999; Igawa et al. 2007; Dong et al. 2008; Han et al. 2016). In some cases, these genes are derived from the pathogens themselves (Thomas et al. 2020).
- Advances in our ability to control protein expression through the use of translation control mediated by upstream open reading frames (uORFs) may allow the more precise control of gene expression and may mitigate the yield penalties associated with the induction of the plant defence response discussed above (Xu et al. 2017; Wang et al. 2024).
- Our improved abilities to perform targeted amplification and sequencing of plant resistance genes have facilitated the wide‐scale identification of resistance genes from crop wild relatives, making it possible to transfer large numbers of *R*‐genes between related species (Witek et al. 2016; Lin et al. 2023). Our ability to insert genes into precise genomic locations via CRISPR‐Cas9 mediated gene insertion (Gao et al. 2020) allows the stacking of multiple *R*‐genes at specific locations that are advantageous for standardised levels of gene expression, for ease of breeding and, from a regulatory perspective, for commercial deregulation.
- PTI mechanisms appear to be largely conserved across higher plants such that PRRs derived from monocots retain their function when introduced into dicots. Because PRR repertoires, and consequent recognitional abilities, vary across different plant species, it is possible to transfer PRRs across plant species to increase their resistance spectrums (Hudson et al. 2024). Furthermore, it is possible to generate novel chimeric PRRs that combine new recognitional specificities with endogenous signalling domains (Albert et al. 2010; Brutus et al. 2010; Kishimoto et al. 2010; Kouzai et al. 2013; Holton et al. 2015; Schwessinger et al. 2015; Thomas et al. 2018; Wu et al. 2019)
- Our current understanding of resistance gene networks (Contreras et al. 2023a) presents the possibility that specific edits can be made in key parts of the network that ‘resurrect’ the functions of multiple previously ‘defeated’ *R*‐genes (Contreras et al. 2023b).
- The control of programmed cell death has been proposed as a promising approach for engineering resistance to necrotrophic diseases for more than 20 years (Dickman et al. 2001), though we still have much to learn (Dickman et al. 2017).
- The realisation that many pathogens exploit specific ‘susceptibility’ genes in the host opens the possibility that these genes can be inactivated using gene‐editing techniques, thus conferring increased resistance (Garcia‐Ruiz et al. 2021).
- Our increasingly sophisticated understanding of the molecular functioning of plant resistance genes allows approaches to the design of bespoke artificial resistance genes designed for the recognition of specific epitopes (Marchal et al. 2022; Kourelis et al. 2023).

## 3 Why Have These Approaches Not Yet Led to Application?

The status of efforts in engineering plant disease resistance through biotechnological approaches has been reviewed numerous times over the years. While, as noted above, the approaches proposed and pursued have become increasingly sophisticated and diverse, one common theme emerges in these reviews:

‘*The combination of these defenses with the added protection provided by expression of potent antifungal proteins promises the future delivery to the grower of an effective arsenal to combat the most important microbial diseases limiting crop production today’* (Salmeron and Vernooij 1998).

‘*Although disease‐resistant transgenic plants or seeds are not yet available commercially, future product development seems likely as our current level of understanding of pathogenesis and plant defence improves’* (Stuiver and Custers 2001).

‘*… Despite the technological advances in developing disease resistance strategies, the evaluation of these transgenic plants for resistance under field conditions has been reported in only a few studies, and the commercialization potential for bacterial and fungal resistance remains to be seen’* (Punja 2006; Wally and Punja 2010).

‘*whilst considerable strategic and practical progress has been made over the last decade, vanishingly few products have been adopted by agriculture…’, ‘It is clear that there are still biological challenges to be met’* (Collinge and Sarrocco 2022).

One could summarise these sentiments as '*We have made a lot of progress; we have not quite got there yet … still, there are reasons for optimism*'.

In some cases, however, a note of despair is expressed:

‘*We have reached a stage where promising biological experimental data must be translated into practical application for the benefit of humanity and the environment. Otherwise, the justification for studying these processes by researchers should be revised’* (Chen et al. 2012).

Many reasons are suggested for this lack of success. These can be divided into ‘societal’ and ‘biological’ impediments. Societal constraints are associated with business and regulation and are common to all commercial transgenic plant traits. These include intellectual property considerations, and the costs required to conduct studies to demonstrate safety to the environment and to consumers. For globally traded crops, the specific studies and datasets required for deregulation are defined by, and can vary considerably between, regional and national authorities. Satisfying these, often complex, requirements is expensive and time‐consuming and the cost of developing a transgenic variety is, consequently, considerable, and has been estimated at $120 million (Anonymous 2022). To recover these costs, the value proposition must be clear and the ultimate market, measured both by acreage and years in market, must be substantial. Only a handful of organisations possess the necessary scientific, legal and marketing expertise and financial resources to achieve this. Despite all this, certain categories of transgenic traits, namely insect and herbicide resistance, have been successfully commercialised in the United States (Dobson 2024) and several other countries.

It is clear therefore that, given the right circumstances, for the right trait with the right economic incentives, these constraints are surmountable. Our purpose here is to ask what the biological and technical constraints to the development of commercial disease resistance traits are, and whether, in the context of the societal constraints, they are likewise surmountable. This is a difficult question to address partly because research and development failures are seldom reported (Matosin et al. 2014). While acknowledging our limited knowledge of commercial research and development, we suggest the following three issues are important:

(1) *Disease resistance and the defence response are complicated*. Despite our burgeoning knowledge, most researchers would concede that we remain ignorant of many of the ways plants resist pathogens. Even in the case of the well‐studied gene‐for‐gene interactions, we do not really know what molecular mechanisms are ultimately responsible for resistance (Jones et al. 2024). Resistance to pathogens is an ancient selection pressure. For example, it is thought likely that sexual reproduction itself evolved at least partly due to a need to reassort host genomes as a response to parasites (Lively 2010). Plants have evolved mechanisms to constantly surveil the environment to determine the optimal division of their resources between biotic defence, growth and reproduction, response to abiotic stress, and so on (Schultz et al. 2013; He et al. 2022). Plant disease pathogenesis strategies are very diverse. A response that confers resistance to one disease may be ineffective against or may even confer susceptibility to another disease, and these responses must therefore be balanced (Glazebrook 2005). Similarly, plants can distinguish between beneficial and harmful microbes and can tailor their response in broadly opposite directions to each category (Thoms et al. 2021). Furthermore, the functioning of most major‐effect R‐proteins is tightly regulated to ensure that they are activated quickly upon pathogen recognition, but that they remain in an inactive state at other times. These regulatory mechanisms involve dozens of genes (Olukolu et al. 2014; Balint‐Kurti 2019), some of which function in concert with specific R‐proteins, while others are more general. Disruption of these regulatory mechanisms (e.g., by introducing an *R*‐gene into a genetic background in which it is not adapted) can lead to an autoimmune phenotype with catastrophic consequences for yield (Tian et al. 2003; Chae et al. 2014).

All these balancing, controlling and distinguishing mechanisms are still poorly understood, but we can confidently suppose that they are extremely complex, are closely integrated with other processes in plants that control vital functions, and have been evolving since the very beginning of eukaryotic life about 2 billion years ago. Therefore, we suggest that the expectation that we can tinker with these processes to increase disease resistance while avoiding negative effects on linked processes affecting traits like yield and other interactions with the environment may, in many cases, prove over‐optimistic. This could be one reason for the often‐observed ‘defence/growth trade‐off’ (He et al. 2022) and why many approaches that are reported to increase resistance in the laboratory and greenhouse without otherwise negative consequences have not been reported to be effective in the field, where the abiotic and biotic environments are much more complex (Chen et al. 2012).

(2) *Plant breeders are good at their jobs; they have a lot to work with, and they are getting better*. Probably as a consequence of evolutionary arms‐race dynamics with a plethora of pathogens over an extended evolutionary period (Maor and Shirasu 2005), components of plant defence mechanisms appear to be evolving rapidly and to show relatively high functional diversity (Tiffin and Moeller 2006; Chen et al. 2012; Tamborski and Krasileva 2020). In most cases and for most crops, plant breeders have been able to work with this diversity to produce high‐yielding varieties that possess adequate resistance to their important disease threats.

From time to time, new threats emerge, and, due to the time required to identify resistance sources and incorporate them into suitable genetic backgrounds, it may take some time for the breeding community to respond. This would appear to be a disadvantage compared to transgenic approaches, where one can insert a resistance gene directly into specific germplasm. However, even in the unlikely event that a resistance gene is available ‘off the shelf’, many elite lines are recalcitrant to transformation and, even if this were not a problem, once a gene is inserted into suitable germplasm and expressed at useful levels, it can take many years to test and deregulate any transgenic event (Bradford et al. 2005). Adequate disease monitoring systems can provide a certain amount of ‘lead time’ for plant breeders to address emerging pathogens. A good example of this was the successful international effort to identify resistance sources for the Ug99 strain of *Puccinia graminis* f. sp. *tritici*, causal agent of wheat stem rust (Aktar‐Uz‐Zaman et al. 2017), and to incorporate them into suitable genetic backgrounds.

In crops that can be conventionally bred, the majority of disease problems have been effectively addressed using conventional breeding approaches. Furthermore, advances in techniques such as genomic selection, high‐throughput phenotyping and double haploidy are providing the breeder with powerful tools to identify useful diversity and to more rapidly incorporate it into elite lines (McGowan et al. 2021). The successful application of plant breeding will often mean that, even if biotechnological approaches yield suitably resistant varieties, their advantages over the conventionally introduced traits may be marginal and not sufficient to justify their high costs of development.

(3) *Disease resistance is a constantly moving target.* The fact that disease resistance traits depend for their commercial effectiveness on the situation of another, often rapidly evolving, species presents problems that we do not face when designing traits that function to mitigate abiotic stresses. Specific disease problems ebb and flow and change as new pathogen strains emerge or are moved around the world and as new crop varieties are introduced. For example, four of the most important maize diseases in terms of yield loss in North America from 2016 to 2019 (Mueller et al. 2020)—anthracnose stalk rot, grey leaf spot, tar spot and Goss's wilt—were of little importance 30–40 years ago. Approaches that confer broad‐spectrum disease resistance may be more likely to be economically viable, yet may be harder to achieve (Wiesner‐Hanks and Nelson 2016).

The most straightforward, and probably most effective, way to introduce disease resistance transgenically is by the transfer of the appropriate *R*‐genes between species. In general, single *R*‐genes are effective against only specific isolates of specific pathogens and are easily defeated when the pathogen modifies or loses the effector that is recognised (Brown 2015; Cowger and Brown 2019; Chen et al. 2021). These issues might be addressed through the simultaneous introduction of multiple *R*‐genes (Brabham et al. 2024), though in this case there are risks that the encoded proteins are not regulated efficiently, leading to low‐level autoimmune phenotypes and yield penalties.

## 4 Lesson and Aspirations

What lessons might we learn from our collective struggles over the last four decades? While this is a difficult and case‐specific question, several general themes occur to us to consider when developing disease‐resistant varieties using biotechnological (transgenic and genome editing) approaches. We also include at the end of this section aspirations in two areas, which, if implemented, may enhance the probability of success in future projects.

- When initiating a project for the production of resistant varieties, our first impulse should be to ask whether conventional breeding approaches are viable. If resistance sources within the primary gene pool are available or if there is an expectation that they can be identified, and if they can be introduced without unacceptable yield drag, it might make sense to concentrate resources on these breeding efforts.
- Transgenic or biotechnological approaches (including gene editing) could be pursued in cases where conventional breeding approaches prove or are likely to prove inadequate. This includes cases like banana, where the crop is largely sterile and hard to genetically improve by conventional means. It might also include cases where no adequate, durable resistance can be identified within primary or secondary gene pools. Examples include diseases like potato late blight or Fusarium head blight of wheat, for which the development of durably resistant varieties has remained elusive over many decades (Haverkort et al. 2016; Buerstmayr et al. 2020).
- It is our opinion that biotechnological approaches that attempt to modify the regulation of the defence response, for instance by altering the expression of important regulatory proteins, are likely to be unsuccessful. This is because, as previously discussed, these processes are likely to be intertwined with the control of other crucial plant processes in ways that we do not appreciate and are hard to separate. For these reasons, we feel that approaches that employ specific targets, such as RNAi‐based approaches that target the expression of specific pathogen genes, or the introduction of new recognitional specificities to the host, may be more effective.
- Several aspects have hindered our ability to understand this field in a systematic way. It would be helpful if studies in this area were standardised with respect to how they are performed and how they are reported. In terms of performance, claims are often made that modified lines show increased resistance while suffering no yield penalty. However, in many of these cases, yield assessments were made in a controlled rather than a field environment, or, if field environments are used, commercial growth conditions (e.g., plant density) are not used and there is little replication across environments (e.g., Zhang et al. 2020b; Li et al. 2024). A recent publication, regarding the reporting of studies that claim genetic modifications conferring yield improvements, urged much more rigorous testing before making these claims (Khaipho‐Burch et al. 2023). It would also be helpful if negative results were reported. In the case of commercial organisations, we wonder if summaries of historical work that are no longer commercially sensitive might be made available. Given the commercial obstacles to cooperation, it may seem unlikely that this will be achievable. However, there are precedents where commercial, government and nonprofit organisations have cooperated towards a common goal (e.g., Isik and McKeand 2019; Rogers et al. 2022).
- The approaches developed by regulatory agencies for reviewing transgenic crops have, historically, been largely based on the review of insect and herbicide resistance traits where overexpression of foreign genes is often used. Engineered disease resistance approaches often use more subtle approaches, and in reviewing them, we urge regulators to take two potentially mitigating factors into account: First, if the introduced genes are from primary or secondary gene pools of targeted crops and, secondly, if the introduced genes are expressed under their endogenous promoters with no to minimal modification, we would argue that they should be seen as less potentially problematic in their efficacy, yield performance and safe uses. In this context, the evolving views of the European Food Safety Authority (EFSA) are positive trends (Naegeli et al. 2020; Molitorisová et al. 2024). With these updated regulatory approaches, the cost of developing resistant varieties by biotechnological approaches would probably become affordable and could be developed by many different players.

## 5 Conclusions

Optimism and the willingness to take risks are essential components in all successful research and development projects. Work in engineered plant disease resistance traits, despite struggles over several decades to produce commercially successful varieties, has been sustained by continually renewed optimism engendered by each new advance in our technological abilities and biological understanding. Nevertheless, after a certain period without significant success, it behoves us to reflect and to ask if there are lessons that can be learned. Ultimately, our feeling is that there are legitimate reasons to think carefully before embarking on a project to engineer disease resistance traits. However, our technological and scientific progress has indeed been rapid, and it seems a matter of time before they bear fruit in this respect. Despite our successes with conventional breeding, plant disease will remain a significant constraint in crop production. So, in cases where conventional approaches are not appropriate or are unsuccessful and where biotechnology‐based approaches seem realistic, we feel efforts to engineer resistance can be justified. In short:

*We have made a lot of progress; we have not quite got there yet… still, there are reasons for optimism*.

## Conflicts of Interest

The authors declare no conflicts of interest.

## Data Availability

Data sharing not applicable to this article as no datasets were generated or analysed during the current study.
